# Logarithmic-Size Post-Quantum Linkable Ring Signatures Based on Aggregation Operations

**DOI:** 10.3390/e28010130

**Published:** 2026-01-22

**Authors:** Minghui Zheng, Shicheng Huang, Deju Kong, Xing Fu, Qiancheng Yao, Wenyi Hou

**Affiliations:** 1 School of Intelligent Science and Engineering, Hubei Minzu University, 39 Xueyuan Road, Enshi 445000, China; mhzheng3@163.com (M.Z.); kong126@outlook.com (D.K.); 202430265@hbmzu.edu.cn (X.F.); 202430331@hbmy.edu.cn (Q.Y.); 202430332@hbmzu.edu.cn (W.H.); 2 School of Cyberspace Security, Sichuan University, Chengdu 610065, China

**Keywords:** linkable ring signature, post-quantum cryptography, aggregate signature, Merkle tree, unforgeability

## Abstract

Linkable ring signatures are a type of ring signature scheme that can protect the anonymity of signers while allowing the public to verify whether the same signer has signed the same message multiple times. This functionality makes linkable ring signatures suitable for applications such as cryptocurrencies and anonymous voting systems, achieving the dual goals of identity privacy protection and misuse prevention. However, existing post-quantum linkable ring signature schemes often suffer from issues such as excessive linear data growth the adoption of post-quantum signature algorithms, and high circuit complexity resulting from the use of post-quantum zero-knowledge proof protocols. To address these issues, a logarithmic-size post-quantum linkable ring signature scheme based on aggregation operations is proposed. The scheme constructs a Merkle tree from ring members’ public keys via a hash algorithm to achieve logarithmic-scale signing and verification operations. Moreover, it introduces, for the first time, a post-quantum aggregate signature scheme to replace post-quantum zero-knowledge proof protocols, thereby effectively avoiding the construction of complex circuits. Scheme analysis confirms that the proposed scheme meets the correctness requirements of linkable ring signatures. In terms of security, the scheme satisfies the anonymity, unforgeability, and linkability requirements of linkable ring signatures. Moreover, the aggregation process does not leak information about the signing members, ensuring strong privacy protection. Experimental results demonstrate that, when the ring size scales to 1024 members, our scheme outperforms the existing Dilithium-based logarithmic post-quantum ring signature scheme, with nearly 98.25% lower signing time, 98.90% lower verification time, and 99.81% smaller signature size.

## 1. Introduction

To address the challenge of achieving strong anonymity in identity authentication without group management, Rivest et al. first proposed a ring signature scheme based on a combination of RSA digital signatures and Rabin trapdoor functions [1]. In this scheme, a signed message can be verified for correctness using only the public keys of ring members, while the actual source of the signature remains untraceable. This capability enables ring signatures to be applied in systems such as cryptocurrencies and anonymous voting to protect identity privacy. Unlike group signatures [2], ring signatures require no group administrator and no complex setup of ring members prior to signing—a signer can spontaneously and independently select the ring members. However, while providing strong anonymity, ring signatures are vulnerable to double-spending attacks. To mitigate this, Liu et al. enhanced the ring signature scheme and introduced the concept of linkable ring signatures [3], which incorporate a linkability feature that allows detection of multiple signatures issued by the same signer, thereby preventing double-spending. Today, linkable ring signatures are widely used in anonymous payment systems. For instance, Lelantus and Triptych employ linkable ring signatures to construct non-interactive anonymous payment schemes with authorized anonymity sets [4,5], utilizing key images and double-tag pairs, respectively, to prevent double-spending by the same signer. Xie et al. [6] proposed a ring signature scheme based on the SM9 cryptographic algorithm. This scheme, constructed using bilinear pairings, exhibits high efficiency under the classical computational model and provides a reference case for applications within specific cryptographic standard frameworks.

However, with the rapid development of quantum computing and the emergence of quantum algorithms such as Shor’s and Grover’s [7,8], conventional ring signature schemes based on classical mathematical hard problems have become vulnerable. Moreover, as anonymous systems scale up, the size and computational overhead required for signing and verification in linkable ring signatures also increase significantly as the application scale expands, resulting in substantial burdens on both storage and computational resources. To address the security threats posed by quantum computing and the scalability challenges in large-scale applications, researchers have begun exploring ring signature schemes based on post-quantum cryptographic primitives. The following literature review will systematically outline the main technical approaches and research advances in this field.

## 2. Literature Review

With the development of quantum computing, ring signature schemes based on traditional mathematical hard problems face serious threats. To address this challenge, researchers have proposed various post-quantum ring signature schemes [9,10,11,12,13,14,15,16], which can be broadly categorized into the following technical approaches:

1. Ring signatures based on the Short Integer Solution (SIS) problem: Kumar et al. have conducted a series of explorations in this direction. They proposed a ring signature scheme that supports the gradual revelation of signers [9], and later designed a convertible, quantum-secure ring signature scheme [10]. These schemes represent beneficial attempts to control the growth of signature sizes, though there remains room for improvement in balancing efficiency and security;
2. Ring signatures based on coding theory: Musa et al. [11] proposed a comprehensive post-quantum signature scheme using matrix groups, demonstrating the potential of multivariate mathematical tools in constructing signatures. However, its efficiency in specialized ring signature scenarios remains moderate;
3. Ring signatures based on lattices: As a mainstream direction in post-quantum cryptography, lattice-based schemes have garnered significant attention due to their solid theoretical foundations and relatively high efficiency. Wen et al. [12] proposed a lattice-based revocable ring signature scheme (LaRRS) tailored for the dynamic characteristics of Vehicular Ad-hoc Networks (VANETs), incorporating a practical member revocation mechanism while ensuring post-quantum security. Gao et al. [13] proposed the first lattice-based linkable ring signature scheme for blockchain privacy protection, achieving quantum security through the hardness of the LWE/SIS problems. Le et al. [14] further designed an identity-based linkable ring signature on lattices, using identity attributes as public keys and achieving strong security under the SIS and Ring-SIS assumptions. Xiong et al. [15] proposed an efficient certificateless signature scheme based on NTRU lattices, which not only resists quantum attacks but also addresses certificate management and key escrow issues. Liu et al. [16] proposed a traceable ring signature scheme based on the NIST-standardized algorithm Dilithium [17], optimizing both signing time and signature size compared to traditional lattice-based schemes. However, in most of the above schemes, signature size and computational overhead still grow linearly with the ring size, limiting their applicability in large-scale ring scenarios.

To break through the efficiency bottleneck of linear growth, researchers have begun exploring sublinear (particularly logarithmic) complexity frameworks. Such schemes typically employ zero-knowledge proofs as the core technique to achieve size compression. Xue et al. [18] proposed a new generic framework for efficient linkable ring signatures and instantiated it with post-quantum primitives, providing a theoretical foundation for efficient constructions. Zhuang et al. [19] integrated threshold signature principles with zero-knowledge proofs to propose a lattice-based linkable threshold ring signature for electronic voting systems. Huiwen et al. [20] constructed a logarithmic-size non-interactive deniable ring signature using Merkle trees and zero-knowledge proofs. Tang et al. [21] designed an identity-based linkable ring signcryption scheme on NTRU lattices.

To provide a more systematic overview and comparison of the key technical approaches and advances in the aforementioned research on post-quantum ring signatures, the main characteristics of these schemes are summarized in Table 1. The table categorizes and synthesizes representative works in terms of methodology, main contributions, and limitations, thereby clearly highlighting the strengths and unresolved issues of each type of scheme.

Based on the analysis in Table 1 and the relevant literature cited above, existing schemes that achieve logarithmic growth commonly rely on complex post-quantum zero-knowledge proof protocols, which require constructing dedicated circuits for underlying hard problems (e.g., LWE, SIS). This significantly increases the implementation complexity and verification overhead of such schemes. Hence, a key common challenge faced by current research is how to realize efficient and fully functional (especially linkable) post-quantum ring signatures without introducing elaborate zero-knowledge proof systems.

To address the aforementioned challenges in constructing post-quantum linkable ring signatures, this paper proposes a logarithmic-size post-quantum linkable ring signature scheme based on aggregation operations, named LAPQ-LRS (Logarithmic-Size Post-Quantum Linkable Ring Signatures Based on Aggregation). The scheme adopts Dilithium, one of the NIST-standardized post-quantum cryptographic algorithms, as the underlying signature primitive for the linkable ring signature, mainly owing to its comprehensive advantages of high computational efficiency, well-structured algorithm design, and good implementation security. Subsequently, while preserving the advantages of the Dilithium post-quantum cryptographic algorithm, the scheme incorporates linkable ring signature operations by organizing ring members’ public keys into a Merkle tree and proposing the Dilithium-based aggregation signature algorithm LAPQ (Logarithmic-Size Post-Quantum on Aggregation) to achieve logarithmic growth. The main research contributions of this work are as follows:

1. A novel logarithmic construction paradigm based on an aggregation signature primitive is proposed for the first time: unlike existing logarithmic schemes that rely on zero-knowledge proofs, this paper designs the first Dilithium-based aggregation signature primitive, LAPQ, and innovatively combines it with a Merkle tree. In this scheme, the public keys of ring members are aggregated layer-by-layer along the Merkle tree path, and the aggregated signature generated by LAPQ itself serves as a valid “membership proof”. As a result, logarithmic growth in signature size is achieved without using any post-quantum zero-knowledge proofs. By incorporating rejection sampling and the Number Theoretic Transform (NTT), the scheme significantly improves computational efficiency while maintaining post-quantum security.
2. Scheme analysis demonstrates the correctness of both the LAPQ and LAPQ-LRS schemes. Furthermore, under the quantum random oracle model, the security of the LAPQ aggregation operation is formally proven. The analysis confirms that the LAPQ-LRS scheme satisfies the essential security properties of ring signatures and linkable ring signatures, including unforgeability, unconditional anonymity, and linkability.
3. Through performance analysis and simulation experiments, the signing time, verification time, and signature size of the proposed LAPQ-LRS scheme were compared with other quantum-attack-resistant ring signature schemes, demonstrating the superior efficiency of our algorithm.

## 3. Design of the LAPQ-LRS Scheme

This section aims to elaborate on the detailed design of the LAPQ-LRS scheme. First, the definitions of various parameters of the scheme are introduced. Next, the design philosophy and architectural rationale are presented. Then, the construction process of the LAPQ aggregation operation is described in detail. Finally, it explains how the LAPQ aggregation mechanism is applied to the construction of the Merkle tree, thereby completing the overall design of the LAPQ-LRS scheme.

### 3.1. Parameter Definitions

The detailed descriptions of the parameters used in this paper are listed in Table 2.

In the signature algorithm, hash computations are performed by hashing strings from set ${\left\{0,1\right\}}^{*}$ into various formatted domains. Here, *CRH* represents a collision-resistant hash function mapped to ${\left\{0,1\right\}}^{384}$. The *ExpandA* function maps $\rho \in {\left\{0,1\right\}}^{256}$ to matrix $A\in R^{k\times l}$. The *ExpendMask* function is used to deterministically generate randomness for the signature scheme, mapping M∥κ to $y\in S_{\gamma_{1}-1}^{l}$. The algorithm employs two distinct functions for high-order and low-order bit separation. The first is $\mathrm{Power}2{\mathrm{Round}}_{q}$, while the second selects α as a divisor of q−1, let $r\in Z_{q}$, and $r=r_{1}\cdot \alpha +r_{0}$. Here, $r_{1}$ and $r_{0}$ represent the high-order and low-order bits of *r*, respectively. It can be noted that in modular *q* arithmetic, the distance between q−1 and 0 is 1, which may cause the magnitude of the remainder $r_{0}$ to increase by 1. This process is referred to as ${\mathrm{Decompose}}_{q}$. Subsequently, the ${\mathrm{MakeHint}}_{q}$ and ${\mathrm{UseHint}}_{q}$ routines are defined; the former generates hint values, and the latter utilizes these hints to recover the high-order bits of the sum. Additionally, the ${\mathrm{HighBits}}_{q}$ and ${\mathrm{LowBits}}_{q}$ routines are introduced, which extract the high-order bits $r_{1}$ and low-order bits $r_{0}$, respectively, from the output of ${\mathrm{Decompose}}_{q}$.

### 3.2. Scheme Design Philosophy and Architectural Rationale

This subsection elaborates on the design philosophy of the LAPQ-LRS scheme, how it is constructed based on Dilithium, and why the Merkle tree structure is adopted, aiming to illustrate the overall construction rationale and technical selection basis of the scheme.

#### 3.2.1. Design Philosophy Based on Dilithium

LAPQ-LRS selects the NIST post-quantum cryptographic standard algorithm Dilithium as the underlying signature primitive, primarily based on the following design philosophies:

- Efficiency and Compactness: Dilithium performs signature and verification operations in the NTT domain, offering high computational efficiency and small signature size, making it suitable for constructing large-scale ring signature systems;
- Avoiding Complex Zero-Knowledge Proof Circuits: Traditional post-quantum ring signatures often rely on zero-knowledge proof protocols to achieve signer anonymity, leading to complex circuits and high overhead. LAPQ-LRS introduces the Dilithium-based aggregation signature operation LAPQ, which directly conceals the signer’s identity during the signing process without constructing zero-knowledge proof circuits. The detailed design of how the LAPQ algorithm is constructed based on Dilithium is shown in Section 3.3;
- Inheriting Post-Quantum Security: The security of Dilithium is based on the Module Learning With Errors (MLWE) and Module Short Integer Solution (MSIS) problems. Since LAPQ-LRS is built upon this foundation, it inherently possesses post-quantum security.

#### 3.2.2. Role and Design Considerations of the Merkle Tree Structure

The adoption of the Merkle tree structure is primarily based on the following considerations:

- Achieving Logarithmic Complexity: By constructing a Merkle tree with ring members’ public keys as leaf nodes, the signing and verification processes involve only nodes along the path from the leaf to the root, resulting in logarithmic complexity and avoiding linear growth;
- Supporting Hierarchical Aggregation: The parent nodes in the Merkle tree are generated by aggregating the public keys of their child nodes through LAPQ, which naturally aligns with the hierarchical concealment requirements of ring signatures;
- Enhancing Privacy Protection: The public key of each node in the tree is the aggregated result of its child nodes’ public keys, and the aggregation process does not leak signer information, providing strong anonymity.

#### 3.2.3. Summary of the Overall Construction Approach

**Figure 1:**
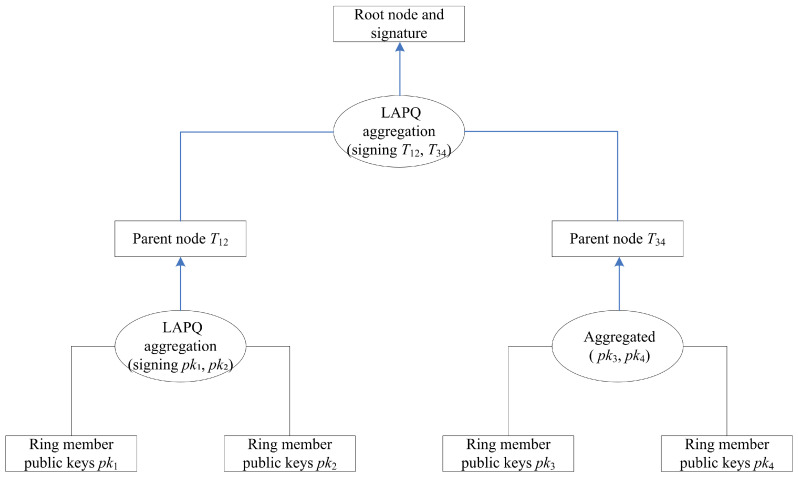
Overall Architecture of the LAPQ-LRS Scheme.

For ease of understanding, Figure 1 illustrates the overall architecture of the scheme.

As illustrated in Figure 1, the overall architecture of the LAPQ-LRS scheme is demonstrated using an example of four nodes. The core idea of the scheme is to organize the public keys of ring members into a Merkle tree structure and construct the tree nodes layer by layer through the LAPQ aggregate signature operation, ultimately producing a ring signature of logarithmic size. The main process includes:

(1) The public keys of the *n* ring members are taken as leaf nodes of the Merkle tree.
(2) Layer-wise aggregation along the signer’s path: at each layer of the Merkle tree, the signer employs the LAPQ aggregation operation to combine the signing node (or the aggregated result from the previous layer) with its sibling node into a parent node, while generating a signature for this aggregation.
(3) Root node generation: the final step yields a root node signature that encompasses the information of all members.
(4) Signature verification: a verifier can validate the correctness of the signature using the corresponding Merkle tree path.

### 3.3. Detailed Design of the LAPQ Scheme

LAPQ is a post-quantum aggregate signature primitive based on Dilithium, which supports aggregating two signers’ public keys into a single verification value and allows signing the aggregated public key. It is suitable for constructing logarithmic-size ring signatures. The LAPQ aggregate signature algorithm is constructed by extending the linear structure and commitment-response mechanism of the Dilithium algorithm. The LAPQ aggregation operation comprises four algorithms: system initialization, key generation, aggregate signature generation, and aggregate signature verification. These are described in detail below.

(1) System Initialization (start(λ)→common): By inputting the security parameter λ, the public parameter set *common* is output.
  $$\lambda =256,common=\left\{\left(\rho ,K\right)\left|\rho \leftarrow {\left\{0,1\right\}}^{256},\right.K\leftarrow {\left\{0,1\right\}}^{256}\right\}$$
(2) Key Generation (key(λ,common)→(sk,pk)): Using the security parameter λ and the public parameter set *common*, the system generates a private key sk; then, the user’s public key pk is derived by inputting sk. All matrix computations are performed in the NTT domain. The NTT transforms convolution operations over the ring $R_{q}$ into pointwise multiplications, reducing the complexity of matrix-vector multiplication from $O\left(n^{2}\right)$ to $O\left(n\log n\right)$. In the concrete implementation, all polynomial sampling, expansion, and linear operations are conducted in the NTT representation, with inverse NTT applied only before output to restore the standard coefficient representation. This optimization is inherited from the CRYSTALS-Dilithium algorithm [17], ensuring that the practical efficiency of the scheme aligns with that of the standardized algorithm. The detailed computation is shown in Algorithm 1.

**Algorithm 1** key
**Input:** λ, *common*
**Output:** pk, sk
**Step 1:** $\left(d_{1},d_{2}\right)\leftarrow S_{\eta }^{l}\times S_{\eta }^{k}$
**Step 2:** $A\in R_{q}^{k\times l}:=\mathrm{ExpendA}\left(\rho \right),\;p:=Ad_{1}+d_{2}$
**Step 3:** $\left(p_{1},p_{2}\right):=\mathrm{Power}2{\mathrm{round}}_{q}\left(p,d\right)$
**Step 4:** $tr\in {\left\{0,1\right\}}^{384}:=\mathrm{CRH}\left(\rho ∥p_{1}\right)$
**Step 5:** return $\left(pk=\left(\rho ,p_{1}\right),sk=\left(tr,d_{1},d_{2},p_{0}\right)\right)$

(3) Aggregate Signature Generation ($\mathrm{LAPQsign}(M,pk_{\pi },pk_{v},sk_{\pi })\to \sigma$): Assume the signer is π and the aggregator is *v*, where subscripts are used to distinguish which node’s public or private key belongs to which party (e.g., $pk_{\pi }$ and $pk_{v}$). Since the public key generated in the key generation phase is split into high and low bits, and the private key also contains distinct values $\left(d_{1},d_{2}\right)\leftarrow S_{\eta }^{l}\times S_{\eta }^{k}$, the notation appends “1” or “2” after the assumed member symbols (such as π and *v*) to represent the separated high/low-bit values and the corresponding different components in the private key. For example, the value represented by π1 (denoted as $p_{\pi 1}$) corresponds to the high-order bits of the original public key generated by signer π.

That is, let the signer’s key pair be denoted as $pk_{\pi }=\left(\rho ,p_{\pi 1}\right)$ and $sk_{\pi }\left(tr_{\pi },d_{\pi 1},d_{\pi 2},p_{\pi 0}\right)$, and the aggregator’s public key is $pk_{v}=\left(\rho ,p_{v1}\right)$. Among these, LAPQ leverages the linear property of Dilithium public keys to aggregate $p_{\pi 1}$ and $p_{v1}$ into a combined public key using the mask $A_{T}\cdot d_{T}$. Subsequently, the scheme executes a Dilithium-like commit-challenge-response process to generate a signature fragment, which includes the response vector, hint value, and aggregated public key. The detailed design of LAPQ aggregate signature generation is presented in Algorithm 2.

(4) Aggregate Signature Verification (LAPQverify(M,σ)→True/TrueFalse.False): The verifier takes the message to be signed *M* and signature fragments σ(s,pk) as inputs and uses Algorithm 3 to verify whether the signature constitutes a valid ring signature for the message. If the verification passes, the output is True, indicating a valid signature; otherwise, the output is False, indicating an invalid signature.

**Algorithm 2** LAPQsign
**Input:** $sk_{\pi }$, $pk_{\pi }$, $pk_{v}$, *M*
**Output:** σ
**Step 1:** $T_{i}=A_{\pi }\cdot d_{\pi 1}+pk_{\pi 1}\cdot 2^{d}$
**Step 2:** $A_{T}\in R_{q}^{k\times l}:=\mathrm{ExpendA}\left(\rho_{T}\right)$, $d_{T}\leftarrow S_{\eta }^{l}\times S_{\eta }^{k}$
**Step 3:** $t=A_{T}\cdot d_{T}+pk_{v_{1}}\cdot 2^{d}$, $T_{i+1}=t+T_{i}$
**Step 4:** $\left(y_{1},y_{2}\right)\in S_{y_{1}-1}^{l}:=\mathrm{ExpendMask}\left(\kappa \|M\right)$, κ:=0
**Step 5:** $\left(w_{1},w_{0}\right)={\mathrm{HighBits}}_{q}\left(A_{T}\left(y_{1}+y_{2}\right),2\gamma_{2}\right)$, $c=H(\left(t,M\right)\|w_{1})$
**Step 6:** $z_{1}=y_{1}+c\cdot d_{T}$, $z_{2}=A_{T}\cdot y_{2}+2c\cdot A_{\pi }\cdot d_{\pi_{1}}+c\cdot p_{v1}\cdot 2^{d}$
**Step 7:** $h:={\mathrm{MakeHint}}_{q}\left(-cp_{\pi_{0}},w_{0}-cd_{\pi_{2}}+cp_{\pi_{0}},2\gamma_{2}\right)$
**Step 8:** $s(\left(z_{1},z_{2}\right),h,c,t)$, $pk=(\rho_{T},T_{i+1})$
**Step 9:** return σ(s,pk)


**Algorithm 3** LAPQverify
**Input:** *M*, σ
**Output:** *True*/*False*
**Step 1:** Check whether $\sigma \left(s,pk\right)\in S_{\eta }^{l}\times S_{\eta }^{k}$ holds. If true, proceed to Step 2; otherwise, return *False*.
**Step 2:** $A_{T}\in R_{q}^{k\times l}:=\mathrm{ExpendA}\left(\rho_{T}\right)$
**Step 3:** $w_{1}^{\prime }:={\mathrm{UseHint}}_{q}\left(h,A_{T}z_{1}+z_{2}-c\cdot T_{i+1},2\gamma_{2}\right)$, $c^{\prime }=H\left(\left(t,M\right)\|w_{1}^{\prime }\right)$
**Step 4:** Check whether $c=c^{\prime }$ is correct. If correct, return *True*; otherwise, return *False*.


### 3.4. Detailed Design of the LAPQ-LRS Scheme

**Figure 2:**
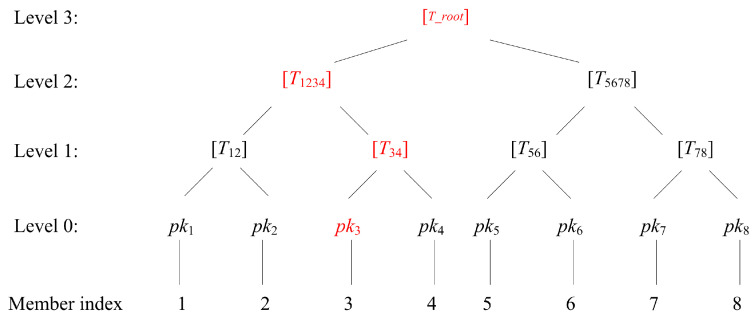
Example diagram of signing path and logarithmic growth.

Based on the LAPQ aggregation signature primitive, the LAPQ-LRS scheme organizes ring member nodes as leaf nodes of a Merkle tree and leverages the LAPQ aggregation operation to construct the complete Merkle tree layer by layer. Figure 2 uses a ring size of 8 as an example, visually illustrating the signing path and how logarithmic growth in signature size is achieved. As shown in Figure 2, assuming the signer is node 3 (corresponding to public key $pk_{3}$), its signing path must include all aggregation proofs from the leaf node to the root node, as detailed below:

- Level 0 → Level 1: Perform LAPQ aggregation on the leaf-node pair $\left(pk_{3},pk_{4}\right)$, generating the parent node $T_{34}$ and the corresponding aggregated signature $s_{1}$.
- Level 1 → Level 2: Perform LAPQ aggregation on the leaf-node pair $\left(T_{34},T_{12}\right)$, generating the parent node $T_{1234}$ and the corresponding aggregated signature $s_{2}$.
- Level 2 → Level 3: $\left(T_{1234},T_{5678}\right)$, generating the parent node T_root and the corresponding aggregated signature $s_{3}$.

Ultimately, the ring signature σ includes the root-node information T_root, along with all $\left[\log_{2}8\right]=3$ signature fragments $\left\{s_{1},s_{2},s_{3}\right\}$ and the corresponding aggregated public-key information in the path, achieving logarithmic growth in the ring signature size as the scale expands. The LAPQ-LRS scheme comprises four algorithms: system initialization, key generation, signature generation, and signature verification. Among these, system initialization and key generation are identical to those in the LAPQ primitive, while signature generation and signature verification are specifically designed for ring signatures based on the LAPQ aggregation operation combined with the Merkle tree construction process described above. The detailed algorithms are presented below, which are described in detail below.

(1) System Initialization (start(λ)→common): By inputting the security parameter λ, the public parameter set common is output. Here λ=256, and $\mathrm{common}=\{\left(\rho ,K\right)|\rho \leftarrow {\left\{0,1\right\}}^{256},K\leftarrow {\left\{0,1\right\}}^{256}\}$.
(2) Key Generation (key(λ,common)→(sk,pk)): This process is identical to that in Dilithium, i.e., it follows the same key generation algorithm as LAPQ. The detailed computation is shown in Algorithm 1.
(3) Aggregate Signature Generation ($\mathrm{sign}(M,P,sk_{\pi })\to \sigma$): The scheme employs the LAPQ aggregate signature operation, with detailed design shown in Algorithm 2. The LAPQ aggregation operation completes the sibling node signing phase, where sibling nodes act as aggregators in the LAPQ operation. The resulting aggregated node in LAPQ becomes the parent node at the next level in the LAPQ-LRS scheme.

To integrate LAPQ aggregation into the Merkle tree construction, the LAPQ-LRS scheme divides the LAPQ process into two phases: parent node public key generation and sibling node signing. These are combined with Merkle tree construction to achieve linkable ring signatures, ensuring that the LAPQ phase occurs during the generation of each subsequent parent node.

The signer takes their private key $sk_{\pi }=\left(tr_{\pi },d_{\pi 1},d_{\pi 2},p_{\pi 0}\right)$, the set of public key vectors *P*, and the message to be signed *M* as inputs, and generates the ring signature σ via Algorithm 4. Here, the signer’s public key is $pk_{\pi }=\left(\rho ,p_{\pi 1}\right)$, the set of ring member public key vectors is $P=(pk_{11},\ldots ,pk_{\pi 1},\ldots ,pk_{n1})$, and the signer’s participation matrix is $A_{\pi }$.

(4) Signature Verification (verify(M,P,σ)→True/TrueFalse.False): To verify the signed message generated in LAPQ-LRS, the verifier takes the message to be signed *M*, the set of public key vectors *P*, and the ring signature $\sigma (\lambda ,s_{1},s_{2},\ldots ,s_{|\log n|+1},pk_{1},pk_{2},\ldots ,pk_{|\log n|+1})$ as inputs. In response to the application of the LAPQ aggregate signature in Algorithm 4, the aggregate verification in LAPQ is modified from Algorithm 3 to Algorithm 5 to verify whether the signature constitutes a valid ring signature for the message. First, check whether the following condition holds $\sigma (\lambda ,s_{1},s_{2},\ldots ,s_{|\log n|+1},pk_{1},pk_{2},\ldots ,pk_{|\log n|+1})$$\in S_{\eta }^{l}\times S_{\eta }^{k}$. If the verification is successful, proceed to check whether condition $pk_{|\log n|+1}=\lambda +\mathrm{P\_sum}\cdot 2^{d}$ is satisfied, where P_sum represents the aggregated vector of ring members. If both conditions are met, execute Algorithm 5. Output True to indicate a valid signature; otherwise, output False to indicate an invalid signature.

**Algorithm 4** sign
**Input:** $sk_{\pi }$, *P*, *M*
**Output:** σ
**Step 1:** $T_{i}=A_{\pi }\cdot d_{\pi 1}+pk_{\pi 1}\cdot 2^{d}$, $\lambda =\alpha =A_{\pi }\cdot d_{\pi 1}$, i=0, v=π
**Step 2: while** len(*P*) >1:
 
//*Parent Node Public Key Generation*
**Step 3:**         $A_{T}\in R_{q}^{k\times l}:=\mathrm{ExpendA}\left(\rho_{T}\right)$, $d_{T}\leftarrow S_{h}^{l}\times S_{h}^{k}$
**Step 4:**         $t=A_{T}\cdot d_{T}+pk_{v\pm 1}\cdot 2^{d}$, $\beta =A_{T}\cdot d_{T}$, α=λ
**Step 5:**         $T_{i+1}=t+T_{i}$, λ=α+β
//*Sibling Node Signing*
**Step 6:**         $\left(y_{1},y_{2}\right)\in S_{\gamma_{1}-1}^{l}:=ExpendMask\left(\kappa ||M\right)$, κ:=0
**Step 7:**         $\left(w_{1},w_{0}\right)=HighBits_{q}\left(A_{T}\left(y_{1}+y_{2}\right),2\gamma_{2}\right),c=H\left(\left(t,M\right)||w_{1}\right)$
**Step 8:**         $z_{1}=y_{1}+c\cdot d_{T}$, $z_{2}=A_{T}\cdot y_{2}+cA_{\pi }\cdot d_{\pi 1}+c\left(T_{i+1}-\lambda -p_{\pi 1}\right)\cdot 2^{d}+c\alpha$
**Step 9:**         $h:=MakeHint_{q}\left(-cp_{\pi 0},w_{0}-cd_{\pi 2}+cp_{\pi 0},2\gamma_{2}\right)$
**Step 10:**         $s_{i+1}\left(\left(z_{1},z_{2}\right),h,c,t\right)$, $pk_{i+1}=\left(\rho_{T},T_{i+1}\right)$
 
//*Merkle Tree Construction*
**Step 11:**          next_level ← []
**Step 12:**          for j from 0 to length(*P*)-1 with step 2:
**Step 13:**                  if j + 1 < length(*P*):
**Step 14:**                         if j = v or j + 1 = v:
**Step 15:**                                append $T_{i+1}$ to next_level
**Step 16:**                          else: append $\left(pk_{j1}+pk_{j2}\right)$ to next_level
**Step 17:**                   **else:** append $pk_{j1}$ to next_level
**Step 18:**           π←π÷2, P← next_level, i←i+1
 
return: $\sigma (\lambda ,s_{1},s_{2},\ldots ,s_{|\log n|+1},pk_{1},pk_{2},\ldots ,pk_{|\log n|+1})$


**Algorithm 5** verify
**Input:** *M*, *P*, σ
**Output:** *True*/*False*
**Step 1:** for i=1,2,…,|logn|+1 do
**Step 2:** Compute matrices $s_{i}$ and $pk_{i}$, and calculate $A_{T}\in R_{q}^{k\times l}:=\mathrm{ExpendA}\left(\rho_{T}\right)$
**Step 3:** $w_{1}^{\prime }:=\mathrm{UseHit}\left(h,A_{T}z_{1}+z_{2}-c\cdot T_{i},2\gamma_{2}\right)$, $c^{\prime }=H\left(\left(t,M\right)\|w_{1}^{\prime }\right)$
**Step 4:** Check whether $c=c^{\prime }$ is correct. If correct, then return *True* else return *False*


## 4. Algorithm Analysis

This section primarily analyzes the correctness and security of the LAPQ aggregation signature and the LAPQ-LRS linkable ring signature scheme. Before proving that the LAPQ and LAPQ-LRS schemes satisfy correctness, the interrelations among the modules in these schemes are first introduced. Since the key generation phase and modules of the LAPQ and LAPQ-LRS schemes are based on the Dilithium signature algorithm, the LAPQ and LAPQ-LRS schemes adhere to Lemmas 1 and 2 from the Dilithium signature algorithm [17]. To facilitate a clearer understanding of the correctness proof process for the LAPQ and LAPQ-LRS schemes, the contents of Lemmas 1 and 2 are presented below.

**Lemma** **1.** *Suppose that q and α are positive integers satisfying q>2α, q≡1modα and α even. Let r and z be vectors of elements in $R_{q}$ where ${∥z∥}_{\infty }\leq \alpha /2$, and let $h,h^{\prime }$ be vectors of bits. Then the $HighBits_{q}$, $MakeHint_{q}$, and $UseHint_{q}$ algorithms satisfy the following properties:*

*1.*  *$UseHint_{q}\left(MakeHint_{q}\left(z,r,\alpha \right),r,\alpha \right)=HighBits_{q}\left(r+z,\alpha \right)$.*
*2.*  *Let $v_{1}=UseHint_{q}\left(h,r,\alpha \right)$. Then $∥r-v_{1}{\cdot \alpha ∥}_{\infty }\leq \alpha +1$. Furthermore, if the number of 1’s in h is ω, then all except at most ω coefficients of $r-v_{1}\cdot \alpha$ will have magnitude at most α/2 after centered reduction modulo q.*
*3.*  *For any $h,h^{\prime }$, if $UseHint_{q}\left(h,r,\alpha \right)=UseHint_{q}\left(h^{\prime },r,\alpha \right)$, then $h=h^{\prime }$.*

**Lemma** **2.** *If ${∥s∥}_{\infty }\leq \beta$ and ∥LowBitsq∥∞≤α/α22−β, then*

$$HighBits_{q}\left(r,\alpha \right)=HighBits_{q}\left(r+s,\alpha \right)$$

### 4.1. Correctness and Security Analysis of the LAPQ Aggregation Signature

LAPQ is a post-quantum aggregation signature primitive based on Dilithium, which aggregates the public-key information of two signers into a single verifiable structure. This section formally defines its security properties and threat model, providing a theoretical foundation for its use as an independent cryptographic component.

#### 4.1.1. Correctness Analysis of LAPQ

**Theorem** **1.** *The LAPQ scheme satisfies the correctness of aggregation signatures.*

For any value output by the “Aggregation Signature Generation” algorithm, when input into the aggregate signature verification algorithm, it always outputs “*True*”. That is, the aggregate signature generated by the signer’s key pair $\left(pk_{\pi },sk_{\pi }\right)$ and any public key $pk_{v}=\left(\rho ,p_{v1}\right)$ on a message *M* must satisfy $\left(LAPQverify\left(M,\sigma \right)\to True\right)$. Based on the above analysis, if the LAPQ scheme satisfies correctness, it necessarily fulfills the following equality:

$$c=c^{\prime }$$

**Proof.**  According to Algorithm 2, the commitment value generated during the signing phase is $c=H\left(\left(t,M\right)\left|\right|w_{1}\right)$. That is, given that the sibling node’s public-key vector *t* and the message *M* are transmitted correctly in each signature fragment, it suffices to prove $w_{1}={w_{1}}^{\prime }$. Assume verification is performed on the signature fragment *s*. It is known that during the aggregate verification phase, ${w_{1}}^{\prime }$ is generated as shown below:

$${w_{1}}^{\prime }:=UseHint_{q}\left(h,A_{T}z_{1}+z_{2}-c\cdot T_{i+1},2\gamma_{2}\right)$$

For $A_{T}z_{1}+z_{2}-c\cdot T_{i+1}$ in the above equation, the following derivation is performed. Here, $T_{i+1}$ is the vector component of the aggregated public key $pk=\left(\rho_{T},T_{i+1}\right)$, with $T_{i+1}=t+T_{i}$, $t=A_{T}\cdot d_{T}+pk_{v1}\cdot 2^{d}$, and $T_{i}=A_{\pi }\cdot d_{\pi 1}+pk_{\pi 1}\cdot 2^{d}$ given as follows:

$$\begin{array}{cc} A_{T}z_{1}+z_{2}-c\cdot T_{i+1} & =A_{T}\left(y_{1}+cd_{T}\right)+\left[A_{T}\cdot y_{2}+2c\cdot A_{\pi }\cdot d_{\pi 1}+c\cdot p_{v1}\cdot 2^{d}\right]-cT_{i+1}\\ \; & =A_{T}\left(y_{1}+y_{2}\right)+cA_{T}\cdot d_{T}+2cA_{\pi }\cdot d_{\pi 1}+c\cdot pk_{v1}\cdot 2^{d}\\ \; & \;-c\left(A_{T}\cdot d_{T}+pk_{v1}\cdot 2^{d}+A_{\pi }\cdot d_{\pi 1}+pk_{\pi 1}\cdot 2^{d}\right)\\ \; & =A_{T}\left(y_{1}+y_{2}\right)+cA_{\pi }\cdot d_{\pi 1}-c\cdot pk_{\pi 1}\cdot 2^{d} \end{array}$$

and the signer π is aware that $A_{\pi }\cdot d_{\pi 1}=p_{\pi 0}+p_{\pi 1}\cdot 2^{d}-d_{\pi 2}$. Hence, the above equation is further derived as follows:

$$\begin{array}{cc} A_{T}z_{1}+z_{2}-c\cdot T_{i+1} & =A_{T}\left(y_{1}+y_{2}\right)+cA_{\pi }\cdot d_{\pi 1}-c\cdot pk_{\pi 1}\cdot 2^{d}\\ \; & =A_{T}\left(y_{1}+y_{2}\right)+c\left(p_{\pi 0}+p_{\pi 1}\cdot 2^{d}-d_{\pi 2}\right)-c\cdot pk_{\pi 1}\cdot 2^{d}\\ \; & =A_{T}\left(y_{1}+y_{2}\right)+c\cdot p_{\pi 0}-c\cdot d_{\pi 2} \end{array}$$

and given the hidden value $h:=MakeHint_{q}\left(-cp_{\pi 0},w_{0}-cd_{\pi 2}+cp_{\pi 0},2\gamma_{2}\right)$, it follows from Lemma 1 that:

$${w_{1}}^{\prime }:=UseHint_{q}\left(h,A_{T}z_{1}+z_{2}-c\cdot T_{i+1},2\gamma_{2}\right)=HighBits_{q}\left(A_{T}\left(y_{1}+y_{2}\right)-cd_{\pi 2},2\gamma_{2}\right)$$

Furthermore, according to Lemma 2, it can be derived that:

$${w_{1}}^{\prime }:=HighBits_{q}\left(A_{T}\left(y_{1}+y_{2}\right)-cd_{\pi 2},2\gamma_{2}\right)=HighBits_{q}\left(A_{T}\left(y_{1}+y_{2}\right),2\gamma_{2}\right)=w_{1}$$

□

Through the above proof, given that the message transmission is correct, it necessarily holds that $c=c^{\prime }$ during the verification phase. Therefore, the LAPQ scheme satisfies the correctness of the aggregation signature.

#### 4.1.2. Security Game Model of LAPQ

A correct and secure aggregation signature should computationally satisfy correctness, and in terms of security, it should possess aggregation unforgeability and aggregation indistinguishability. The correctness of the LAPQ scheme has already been proven above. This subsection will formalize the proofs of unforgeability and aggregation indistinguishability for the LAPQ scheme through a security game between a challenger **C** and an adversary **E**. Moreover, as current quantum algorithms cannot effectively solve the Module Learning With Errors (MLWE) and the Module Short Integer Solution (MSIS) problems based on lattices, the following assumptions are reasonable. The scheme employs an oracle-based security model to analyze the LAPQ scheme. The adversary **E** can access the following three oracles:

- Join Oracle JO: generates a new user key pair $\left(pk_{i},sk_{i}\right)$, returns the public key $pk_{i}$ to the adversary, and secretly stores the private key $sk_{i}$ by the challenger;
- Corruption Oracle CO: takes a public key $pk_{i}$ as input and outputs the corresponding private key $sk_{i}$. In the security game, at least one honest user must be retained for the security challenge;
- Aggregation Signature Oracle ASO: takes $\left(M,pk_{i},pk_{j},sk_{i}\right)$ as input and outputs an aggregated signature σ, where $\left(pk_{i},sk_{i}\right)$ is the signer’s key pair, $pk_{j}$ is the public key to be aggregated, and *M* is the message to be signed.

The adversary may adaptively query these oracles a polynomial number of times. Based on this model, the following security properties are formally defined:

**Theorem** **2.** *If the Module Learning With Errors (MLWE) problem and the Module Short Integer Solution (MSIS) problem are hard, then the LAPQ scheme satisfies aggregation unforgeability.*

**Proof.**  Consider the following security game model. The adversary **E** can adaptively query the oracles JO, CO, and ASO a polynomial number of times. In the game, the adversary is not allowed to query the aggregation signature on the target message *M* under the target public key pair $\left(pk_{\pi },sk_{\pi }\right)$. If there exists an adversary **E** that can forge a valid aggregated signature message with non-negligible probability ε via the oracles JO, CO, and ASO, then a challenger **C** can solve the MLWE and MSIS problems with approximately the same probability ε. First, the challenger **C** generates the system parameters *common*, produces a public key $pk_{\pi }=\left(\rho ,p_{\pi 1}\right)$ and a private key $sk_{\pi }\left(tr_{\pi },d_{\pi 1},d_{\pi 2},p_{\pi 0}\right)$, signs the message *M*, and obtains the signature result $\sigma \left(s,pk\right)$ and $s\left(\left(z_{1},z_{2}\right),h,c,t\right)$. Subsequently, the adversary **E** constructs the forged signature through the following operations.

(1) For any signature fragment of a signed message *M*, the adversary **E** may query the oracles JO, CO, and ASO multiple times. It randomly assigns the correct $\left(A^{\prime },p^{\prime }\right)$.
(2) With probability ε, the adversary **E** outputs any signature fragment $\sigma \left(s^{\prime },pk^{\prime }\right)$ of the forged signature message $M^{\prime }$ along with the forged signature $s^{\prime }\left(\left({z_{1}}^{\prime },{z_{2}}^{\prime }\right),h^{\prime },c^{\prime },t^{\prime }\right)$, $pk^{\prime }=\left({\rho_{T}}^{\prime },{T_{i+1}}^{\prime }\right)$.
(3) If $\left(LAPQverify\left(M^{\prime },\sigma^{\prime }\right)\to True\right)$ holds, the adversary **E** wins.

If the adversary **E** wins, it necessarily holds that ${∥\left({z_{1}}^{\prime },{z_{2}}^{\prime }\right)∥}_{\infty }\leq \gamma_{1}-\beta$, and $H\left(\left(t,M\right)||UseHint_{q}\left(h^{\prime },A^{\prime }{z_{1}}^{\prime }+{z_{2}}^{\prime }-c^{\prime }\cdot {T_{i+1}}^{\prime },2\gamma_{2}\right)\right)=c^{\prime }$. Then, if the signature is legally valid, it must satisfy $\left({z_{1}}^{\prime },{z_{2}}^{\prime }\right)=\left(z_{1},z_{2}\right)$. Consequently, the adversary **E** has obtained the private key $sk_{\pi }\left(tr_{\pi },d_{\pi 1},d_{\pi 2},p_{\pi 0}\right)$, thereby solving the underlying MLWE and MSIS problems. However, the Module Learning With Errors (MLWE) and the Module Short Integer Solution (MSIS) problems are hard. Therefore, the LAPQ scheme possesses unforgeability. □

**Theorem** **3.** *If the Module Learning With Errors (MLWE) problem and the Module Short Integer Solution (MSIS) problem are hard, then the LAPQ scheme satisfies aggregation indistinguishability.*

**Proof.**  Given two public key pairs $pk_{\pi }=\left(\rho ,p_{\pi 1}\right)$ and $pk_{v}=\left(\rho ,p_{v1}\right)$, along with an LAPQ aggregated signature $\sigma \left(s,pk\right)$, the adversary **E** cannot distinguish which public key generated $\sigma \left(s,pk\right)$. The security game model is as follows:

(1) The adversary **E** chooses two sets of public keys $pk_{\pi }=\left(\rho ,p_{\pi 1}\right)$ and $pk_{v}=\left(\rho ,p_{v1}\right)$.
(2) The challenger **C** randomly selects $a\leftarrow \left\{\pi ,v\right\}$.
(3) The challenger **C** computes $\sigma \leftarrow LAPQsign\left(M,pk_{b},pk_{b},sk_{b}\right)$.
(4) After receiving σ, the adversary **E** guesses $a^{\prime }$. If $a^{\prime }=a$ holds, the adversary wins.

If the adversary **E** can distinguish the aggregation source with a non-negligible advantage, it would imply that **E** can determine which public key generated the signature σ. For the signed message σ, Algorithm 2 shows that the parameters containing public-key information in the signature message are $z_{2}=A_{T}\cdot y_{2}+2c\cdot A_{\pi }\cdot d_{\pi 1}+c\cdot p_{v1}\cdot 2^{d}$ and $t=A_{T}\cdot d_{T}+pk_{v1}\cdot 2^{d}$. If the adversary **E** can unambiguously determine from which public key the signature message originates, then **E** can solve for the public key $pk_{v1}$ from $z_{2}$ and *t*, thereby solving the underlying MLWE and MSIS problems. However, the Module Learning With Errors (MLWE) and the Module Short Integer Solution (MSIS) problems are hard. Consequently, the LAPQ scheme possesses aggregation indistinguishability. □

### 4.2. Correctness and Security Analysis of the LAPQ-LRS Ring Signature

#### 4.2.1. Correctness Analysis of LAPQ-LRS

**Theorem** **4.** *The LAPQ-LRS scheme satisfies the correctness requirement for linkable ring signatures.*

For any value output by the “Signature Generation” algorithm, when input into the signature verification algorithm or the linkable signature verification algorithm, the result is always “*True*”. Assuming the signature $\sigma (\lambda ,s_{1},\ldots ,s_{|\log n|+1},pk_{1},\ldots ,pk_{|\log n|+1})$ from signer π is entirely correct, the following correctness conditions must be satisfied:

(1) Whether the signature message in the LAPQ-LRS scheme satisfies the same computational domain, as follows.
  $$\sigma \left(\lambda ,s_{1},s_{2},\ldots ,s_{\left|\log n\right|+1},pk_{1},pk_{2},\ldots ,pk_{\left|\log n\right|+1}\right)\in S_{\eta }^{l}\times S_{\eta }^{k}$$

**Proof.**  In Algorithm 2, $\alpha =A_{\pi }\cdot d_{\pi 1}$ or α=α+β holds, and with $A_{T},\;A_{\pi }\in R_{q}^{k\times l}$ and $d_{T},\;d_{\pi }\leftarrow S_{\eta }^{l}\times S_{\eta }^{k}$, thus $\alpha \in S_{\eta }^{l}\times S_{\eta }^{k}$ follows. For different signature fragments $s_{i}\left(z_{1},z_{2},h,c,t\right)$, all are mapped from the NTT domain to $S_{\eta }^{l}\times S_{\eta }^{k}$, hence $s_{i}\leftarrow S_{\eta }^{l}\times S_{\eta }^{k}$ holds. The public key vector $pk_{i}$ in verification public key $T_{i+1}=t+T_{i}$ is essentially equivalent to $T_{i}=A_{\pi }\cdot d_{\pi 1}+pk_{\pi 1}\cdot 2^{d}$, and both are mapped from the NTT domain to $S_{\eta }^{l}\times S_{\eta }^{k}$. Therefore, the signature message belongs to the same computational domain. □

(2) Whether the root public key in the LAPQ-LRS scheme is generated with the participation of ring members.
  $$pk_{\left|\log n\right|+1}=\lambda +\mathrm{P\_sum}\cdot 2^{d}$$

**Proof.**  From Algorithm 2, it follows that $pk_{|\log n|+1}=\alpha +\beta +\left(pk_{v}+pk_{v\pm 1}\right)\cdot 2^{d}$. Based on the algorithm analysis, $pk_{v}$ represents the set of public key vectors of two child nodes with itself as the parent node, and by extension down to the leaf nodes, it corresponds to 1/2 of the ring member vector public key set. Similarly, $pk_{v\pm 1}$, extending to the leaf nodes, represents another distinct 1/2 of the ring member vector public key set, and λ=α+β holds. Therefore, the root node public key $pk_{|\log n|+1}$ equals the sum of the aggregated public key vectors of all ring member plus λ. The equation $pk_{|\log n|+1}=\lambda +\mathrm{P\_sum}\cdot 2^{d}$ is thus satisfied. □

(3) Whether the commitment value generated at each layer of the LAPQ-LRS scheme equals the commitment value produced during the signing phase.
  $$c=c^{\prime }$$

**Proof.**  According to Algorithm 2, the commitment value generated during the signing phase is $c=H\left(\left(t,M\right)\|w_{1}\right)$. That is, under the condition that each distinct signature fragment transmits the sibling node’s public key vector *t* and the message *M* correctly, it suffices to prove $w_{1}=w_{1}^{\prime }$. Assume the signature fragment $s_{i}$ is subjected to verification. It is known that the signing phase generates $w_{1}^{\prime }$ as follows:

$${w_{1}}^{\prime }:=UseHint_{q}\left(h,A_{T}z_{1}+z_{2}-c\cdot T_{i},2\gamma_{2}\right)$$

For $A_{T}z_{1}+z_{2}-c\cdot T_{i}$ in the above equation, the derivation is as follows, where $T_{i}$ is the vector component corresponding to the public key $s_{i}$:

$$A_{T}z_{1}+z_{2}-c\cdot pk=A_{T}\left(y_{1}+cd_{T}\right)+\left[A_{T}\cdot y_{2}+cA_{\pi }\cdot d_{\pi 1}+c\left(T_{i}-\lambda -p_{\pi 1}\right)\cdot 2^{d}+c\alpha \right]-cT_{i}$$

where the public key vector is $T_{i}=\alpha +\beta +pk\cdot 2^{d}+pk_{\pi 1}\cdot 2^{d}$, with λ=α+β and $\beta =A_{T}\cdot d_{T}$. For any public key $T_{i}$, algorithm analysis shows that pk represents the aggregated set of leaf node public keys along all paths excluding the signer’s own public key $pk_{\pi 1}$. The derivation is as follows:

$$\begin{array}{cc} A_{T}z_{1}+z_{2}-c\cdot pk & =A_{T}\left(y_{1}+cs_{T}\right)+\left[A_{T}\cdot y_{2}+cA_{\pi }\cdot d_{\pi 1}+c\left(T_{i}-\lambda -p_{\pi 1}\right)\cdot 2^{d}+c\alpha \right]-cT_{i}\\ \; & =A_{T}\left(y_{1}+y_{2}\right)+cA_{T}\cdot d_{T}+cA_{\pi }\cdot d_{\pi 1}-c\beta -cp_{\pi 1}\cdot 2^{d}\\ \; & =A_{T}\left(y_{1}+y_{2}\right)+cA_{\pi }\cdot d_{\pi 1}-cp_{\pi 1}\cdot 2^{d} \end{array}$$

and the signer π is aware of $A_{\pi }\cdot d_{\pi 1}=p_{\pi 0}+p_{\pi 1}\cdot 2^{d}-d_{\pi 2}$. Thus, the above equation can be further derived as follows:

$$\begin{array}{cc} A_{T}z_{1}+z_{2}-c\cdot pk & =A_{T}\left(y_{1}+y_{2}\right)+c\left(p_{\pi 0}+p_{\pi 1}\cdot 2^{d}-d_{\pi 2}\right)-cp_{\pi 1}\cdot 2^{d}\\ \; & =A_{T}\left(y_{1}+y_{2}\right)+cp_{\pi 0}-cd_{\pi 2} \end{array}$$

and given the hidden value $h:=MakeHint_{q}\left(-cp_{\pi 0},w_{0}-cd_{\pi 2}+cp_{\pi 0},2\gamma_{2}\right)$, it follows from Lemma 1 that:

$${w_{1}}^{\prime }:=UseHint_{q}\left(h,A_{T}z_{1}+z_{2}-c\cdot T_{i},2\gamma_{2}\right)=HighBits_{q}\left(A_{T}\left(y_{1}+y_{2}\right)-cd_{\pi 2},2\gamma_{2}\right)$$

Furthermore, according to Lemma 2, it can be derived that:

$${w_{1}}^{\prime }:=HighBits_{q}\left(A_{T}\left(y_{1}+y_{2}\right)-cd_{\pi 2},2\gamma_{2}\right)=HighBits_{q}\left(A_{T}\left(y_{1}+y_{2}\right),2\gamma_{2}\right)=w_{1}$$

Through the above proof, given that the message transmission is correct, it necessarily follows that $c=c^{\prime }$ holds during the verification phase. □

#### 4.2.2. Security Game Model of LAPQ-LRS

A correct and secure linkable ring signature should computationally satisfy correctness, and in terms of security, it should possess unforgeability, anonymity, and linkability. The correctness of the LAPQ-LRS scheme has already been proven above. This subsection will formalize the proofs of unforgeability, anonymity, and linkability for the LAPQ-LRS scheme through a security game between a challenger **C** and an adversary **E**. Moreover, as current quantum algorithms cannot effectively solve the Module Learning With Errors (MLWE) and the Module Short Integer Solution (MSIS) problems based on lattices, the following assumptions are reasonable. The scheme employs an oracle-based security model to analyze the LAPQ-LRS scheme. The adversary E can access the following three oracles:

- Join Oracle JO: generates a new user key pair $\left(pk_{i},sk_{i}\right)$, returns the public key $pk_{i}$ to the adversary, and secretly stores the private key $sk_{i}$ by the challenger;
- Corruption Oracle CO: takes a public key $pk_{i}$ as input and outputs the corresponding private key $sk_{i}$. In the unforgeability security game, all users on the target ring *R* cannot be corrupted. In the anonymity security game, the honest challenge user(s) cannot be corrupted. In the linkability security game, at least two distinct honest users must be preserved;
- Ring Signature Oracle SO: takes $\left(sk_{i},P,M\right)$ as input and outputs a ring signature σ, where $sk_{i}$ is the signer’s private key, *P* is the set of public keys, and *M* is the message to be signed.

The adversary may adaptively query the above oracles a polynomial number of times. Based on this model, the following security properties are formally defined:

**Theorem** **5.** *If the Module Learning With Errors (MLWE) and Module Small Integer Solution (MSIS) problems are hard, then the LAPQ-LRS scheme satisfies unforgeability.*

**Proof.**  If there exists an adversary E capable of forging a valid linkable ring signature message with non-negligible probability ε through oracles JO, CO, and SO, then a challenger C can solve the MLWE and MSIS problems with approximate probability ε. First, the challenger C generates the system parameters *common*, produces a public key $pk=(\rho ,p_{1})$ and a private key $sk=(tr,d_{1},d_{2},p_{0})$, and signs the message *M*, resulting in signature $\sigma \left(\lambda ,s_{1},s_{2},\ldots ,s_{|\log n|+1},pk_{1},pk_{2},\ldots ,pk_{|\log n|+1}\right)$. The adversary E then constructs the forged signature through the following operations.

(1) For any signature fragment of a signed message *M*, adversary E can query oracles JO, CO, and SO multiple times. It randomly assigns the correct $\left(A^{\prime },p^{\prime }\right)$.
(2) With probability ε, adversary E outputs any signature fragment $\sigma \left(s_{i},pk_{i}\right)$ of the message *M* to be signed, along with a forged signature
  $$s^{\prime }\left({c_{1}}^{\prime },{c_{2}}^{\prime },\ldots ,{c_{n}}^{\prime },\left({z_{11}}^{\prime },{z_{12}}^{\prime }\right),\left({z_{21}}^{\prime },{z_{22}}^{\prime }\right)\ldots ,\left({z_{n1}}^{\prime },{z_{n2}}^{\prime }\right),{h_{1}}^{\prime },{h_{2}}^{\prime },\ldots ,{h_{n}}^{\prime }\right)$$

Here, ${∥\left({z_{\pi 1}}^{\prime },{z_{\pi 2}}^{\prime }\right)∥}_{\infty }\leq \gamma_{1}-\beta$ must hold, and given $H(\left(t_{i},M\right)\|UseHint_{q}(h^{\prime },A^{\prime }{z_{\pi 1}}^{\prime }$ + ${z_{\pi 2}}^{\prime }-c^{\prime }\cdot {T_{i}}^{\prime },2\gamma_{2}))={c_{\pi }}^{\prime }$, if the signature is legally valid, then $\left({z_{\pi 1}}^{\prime },{z_{\pi 2}}^{\prime }\right)=\left(z_{1},z_{2}\right)$ must necessarily hold. This implies that adversary E has obtained the private key $sk_{\pi }=\left(tr_{\pi },d_{\pi 1},d_{\pi 2},p_{\pi 0}\right)$, thereby solving the underlying MLWE and MSIS problems. However, since the Module Learning With Errors (MLWE) and Module Small Integer Solution (MSIS) problems are hard, the LAPQ-LRS scheme satisfies unforgeability. □

**Theorem** **6.** *The LAPQ-LRS scheme satisfies statistical anonymity.*

**Proof.**  Using the game-hopping technique, it is proven that under the hardness assumption of MLWE, the adversary cannot distinguish the real signer with a non-negligible advantage. Consider the following sequence of games:

1. Game 0 (Real Anonymity Game):

The challenger **C** generates the system parameters *common* and *n* key pairs $\left\{\left(pk_{i},sk_{i}\right)\right\}$ and sends the set of public keys $P\left(pk_{1},\ldots ,pk_{\pi },\ldots ,pk_{n}\right)$ to the adversary **E**. The adversary may adaptively query the oracles JO, CO, and SO. In the challenge phase, the adversary selects a ring *R* and two uncorrupted users $\left(i_{0},i_{1}\right)$. The challenger randomly chooses $b\leftarrow \left\{0,1\right\}$, uses $sk_{ib}$ to generate the ring signature σ, and sends it to the adversary. The adversary outputs a guess $b^{\prime }$. Denote the adversary’s advantage as:

$${\mathrm{Adv}}_{0}=\left|\mathrm{Pr}\left[b^{\prime }=b\right]-1/2\right|$$

2. Game 1 (Simulated Signature Game):

The real algorithm is replaced by a simulation algorithm that does not rely on the private key $sk_{\pi }$:

(1) Randomly choose $\left(y_{1},y_{2}\right)\in S_{\gamma_{1}-1}^{l}$;
(2) Compute $w_{1}=HighBits_{q}\left(A_{T}\left(y_{1}+y_{2}\right),2\gamma_{2}\right)c=H\left(\left(t,M\right)\left|\right|w_{1}\right)$;
(3) Set $z_{1}=y_{1}$ and $z_{2}=A_{T}y_{2}$;
(4) Use rejection sampling so that the distribution of $\left(z_{1},z_{2}\right)$ is statistically close to that of a real signature.

Due to the rejection sampling property of Dilithium, the statistical distance between the simulated signature and the real signature is negligible:

$$\left|{\mathrm{Adv}}_{1}-{\mathrm{Adv}}_{0}\right|\leq {\mathrm{negl}}_{1}\left(\lambda \right)$$

3. Game 2 (MLWE Replacement Game):

Replace $A_{T}d_{T}$ in the signature components with MLWE samples. Specifically, for each aggregation layer $t=A_{T}\cdot d_{T}+pk_{v\pm 1}\cdot 2^{d}$, replace $A_{T}d_{T}$ with a sample drawn from either the MLWE distribution or the uniform distribution. If the MLWE problem is hard, the adversary cannot distinguish:

$$\left|{\mathrm{Adv}}_{2}-{\mathrm{Adv}}_{1}\right|\leq {\mathrm{Adv}}^{\mathrm{MLWE}}\left(\lambda \right)$$

4. Game 3 (Ideal Anonymity Game): All signature components are uniformly random values. At this point, the distribution of the signature is completely independent of the signer’s identity, and the adversary can only guess randomly:
  $${\mathrm{Adv}}_{3}=\frac{1}{2}$$

In Game 2, the adversary must identify the signer’s path in a Merkle tree of height $h=\left\lceil \log n\right\rceil$. At each layer of the aggregation operation, the parent node’s public key $T_{i+1}$ is generated by aggregating the public keys of its two child nodes. Owing to the indistinguishability of the computation of the $A_{T}d_{T}$ term, the adversary’s probability of correctly identifying the signer’s path at a single layer is upper-bounded by:

$$\mathrm{Pr}\leq \frac{1}{2}+{\mathrm{negl}}^{\prime }\left(\lambda \right)$$

Each layer is guaranteed conditional independence by independent randomness. According to the Chernoff bound, the probability that the adversary correctly identifies the entire path satisfies:

$$\mathrm{Pr}\leq \frac{1}{2^{h}}+h\cdot {\mathrm{negl}}_{2}\left(\lambda \right)\leq \frac{1}{n}+O\left(\log n\right)\cdot \mathrm{negl}\left(\lambda \right)$$

Combining the upper bounds on advantage from the games above and considering the differences between the games, the adversary’s overall advantage satisfies:

$${\mathrm{Adv}}^{Anon}\leq \frac{1}{n}+{\mathrm{Adv}}^{MLWE}\left(\lambda \right)+\mathrm{negl}\left(\lambda \right)$$

where the term 11nn decreases as the ring size *n* increases, ${\mathrm{Adv}}^{MLWE}\left(\lambda \right)$ is negligible based on the hardness assumption of MLWE, and $\mathrm{negl}\left(\lambda \right)$ is a negligible term resulting from statistical distance. Hence, the adversary’s advantage is negligible under the security parameter λ, and the LAPQ-LRS scheme satisfies statistical anonymity. □

**Theorem** **7.** *If the Module Learning With Errors (MLWE) problem is hard, then the LAPQ-LRS scheme satisfies linkability.*

**Proof.**  The linkability of the LAPQ-LRS scheme is demonstrated through a game between adversary E and challenger C in the oracle model, under the assumption that the Module Learning With Errors (MLWE) problem is hard.

(1) The challenger C generates the system parameters common and sends them to the adversary E.
(2) The adversary E repeatedly queries the oracle JO using the system parameters *common* to obtain the public key set $P\left(pk_{11},\ldots ,pk_{\pi 1},\ldots ,pk_{n1}\right)$. By querying the corruption oracles $CO(pk_{\pi_{1}})$ and 1≤π≤n, it acquires the private key $sk_{\pi }$ corresponding to $pk_{\pi_{1}}$. The adversary E then sends the public key set *P*, the message to be signed *M*, and the private key $sk_{\pi }$ to the challenger C to generate the signature $\sigma (\lambda ,s_{1},s_{2},\ldots ,s_{|\log n|+1},pk_{1},pk_{2},\ldots ,pk_{|\log n|+1})$.
(3) E outputs a valid signature $\sigma^{\prime }\left(\lambda^{\prime },s_{1}^{\prime },s_{2}^{\prime },\ldots ,{s_{|\log n|+1}}^{\prime },{pk_{1}}^{\prime },{pk_{2}}^{\prime },\ldots ,{pk_{|\log n|+1}}^{\prime }\right)$ that differs from σ by querying the oracle.

From the game between challenger C and adversary E described above, it can be concluded that $\lambda \neq \lambda^{\prime }$ holds. If $\lambda :=\alpha +\beta =A\cdot d:\neq {Q_{\pi }}^{\prime },\sigma \neq \sigma^{\prime }$ is satisfied, where A·d represents the aggregation of different matrices and random temporary keys during the signing phase, then adversary E has successfully generated another valid linkable ring signature $\sigma^{\prime }$ that passes challenger C’s verification while knowing only one private key $sk_{\pi }$. As established by Theorem 2, the LAPQ-LRS scheme satisfies unforgeability, which implies that $\lambda =\lambda^{\prime }$ must hold. Therefore, the LAPQ-LRS scheme achieves linkability. □

### 4.3. Security Characteristics and Threat Mitigation

Based on the theoretical proofs above, the LAPQ-LRS scheme not only fulfills the core security properties of ring signatures and linkable ring signatures (unforgeability, anonymity, and linkability), but its unique design also delivers a set of well-defined security characteristics and practical threat-mitigation capabilities. These properties are built not only on the overall architecture of the LAPQ-LRS scheme but also benefit from the strong security attributes of its underlying primitive—the LAPQ aggregation scheme primitive—namely, aggregation unforgeability and aggregation indistinguishability. This subsection aims to clarify these characteristics and explain how the scheme addresses specific network security threats.

#### 4.3.1. Core Security Characteristics

Post-Quantum Security: The underlying hard problems of the scheme are the Module Learning With Errors (MLWE) and the Module Short Integer Solution (MSIS) problems. Under the quantum random oracle model, these problems are widely believed to resist known quantum attacks, thereby ensuring the long-term security of the scheme in the era of quantum computing.

Unconditional Anonymity: As shown in Theorem 6, even an adversary with unbounded computational power cannot determine the true identity of the signer with a probability higher than random guessing. This provides participants with the strongest privacy guarantee.

Unforgeability and Linkability: Theorem 5 and Theorem 7, respectively, prove that an adversary cannot forge a valid signature without knowledge of any private key (unforgeability), and cannot generate two valid ring signatures for the same message that are unlinkable while holding only one private key (linkability). Together, these constitute the foundation for preventing identity impersonation and double-spending (double-spend attacks).

Strong Security of the Aggregation Signature Primitive: The security of the LAPQ-LRS scheme is rooted in two key properties of the LAPQ aggregation signature primitive: aggregation unforgeability and aggregation indistinguishability.

- Aggregation Unforgeability: Under the assumption that the MLWE/MSIS problems are hard, even if the adversary obtains aggregated signatures of multiple users on multiple sets of messages, it cannot forge a new, valid aggregated signature. This guarantees the authenticity and non-tamperability of each parent-node public key (generated by aggregation) in the Merkle tree;
- Aggregation Indistinguishability: Given an aggregation result, any polynomial-time adversary cannot distinguish which specific pair of original keys produced the result. This property is the basis for achieving Theorem 6; it ensures that during the construction of the Merkle tree, the aggregated parent-node public key does not leak information that allows tracing back to or inferring the identity of its child nodes (i.e., specific ring members), thereby structurally concealing the signer’s identity.

#### 4.3.2. Mitigation of Network Security Threats

The design of the LAPQ-LRS scheme directly provides mitigation measures against the following real-world network security threats:

- Mitigation of Long-Term Threats from Quantum Attacks: Compared to ring signature schemes based on traditional number-theoretic hard problems (e.g., large integer factorization, discrete logarithm), LAPQ-LRS can resist quantum attacks such as Shor’s algorithm. This ensures that after quantum computers mature, anonymous systems relying on this scheme (e.g., privacy-oriented cryptocurrencies, anonymous voting) will not suddenly lose confidentiality and integrity, thereby achieving forward-looking security for the system;
- Prevention of “Double-Spending/Voting” Attacks in Anonymous Systems: This is the core problem addressed by linkable ring signatures. Through the linkability property, the system can publicly detect multiple signatures generated by the same private key on the same message without revealing the signer’s identity. This effectively prevents malicious users from double-spending the same currency in anonymous payment systems or casting multiple votes in anonymous voting systems, thereby preserving the fairness and consistency of the system;
- Resistance to Tracking and De-anonymization Attacks Targeting Signer Identity: The scheme’s unconditional anonymity and the aggregation structure based on the Merkle tree—particularly the security guarantee provided by aggregation indistinguishability—ensure that even if an adversary can monitor all network communications and obtain the entire set of public keys, it cannot link different signatures or infer the signer’s identity by analyzing signature content or intermediate aggregated public keys. This protects user identity security during participation in sensitive activities (e.g., whistleblowing, political voting, private transactions).

In summary, the LAPQ-LRS scheme not only meets rigorous security definitions theoretically, but its design (avoidance of complex ZKPs), logarithmic-scale efficiency characteristics, and the strong security foundation provided by the underlying LAPQ aggregation primitive (aggregation unforgeability and indistinguishability) also enable it to more robustly mitigate comprehensive threats from quantum computing, protocol abuse, identity tracking, and implementation flaws in practical deployments. It thus offers a reliable cryptographic foundation for building the next generation of secure, efficient, and scalable anonymous network systems.

## 5. Performance Analysis and Simulation Experiments

### 5.1. Performance Analysis

This subsection will systematically compare and analyze the performance of the proposed LAPQ-LRS scheme with the non-post-quantum ring signature scheme from [6], as well as the state-of-the-art quantum-attack-resistant ring signature schemes from [9,16], along two dimensions: signing/verification time and signature size. A summary of the performance analysis is then provided.

Based on theoretical analysis, assume the number of ring members in the LAPQ-LRS scheme is n. Taking the LAPQ-LRS scheme as an example, the signing time is primarily distributed across three stages in Algorithm 2: parent node public key generation, sibling node signing, and Merkle tree construction. Algorithm analysis indicates that the parent node public key generation stage is equivalent to generating a new public key via Algorithm 1, with a time consumption denoted as $T_{key}$. The sibling node signing stage differs from the Dilithium signing stage by the addition of a response value, whose time consumption equals that of a verification stage. Let the Dilithium signing time be $T_{Dsign}$ and the LAPQ-LRS verification stage time be $T_{Dverify}$; thus, the sibling signing stage time is $T_{Dsign}+T_{Dverify}$. The Merkle tree construction time equals the time required to aggregate all public keys, denoted as $T_{sum}$. Due to the Merkle tree structure, the number of executions for the parent node public key generation and sibling node signing stages is logn. Therefore, the total signing time is $T_{sign}=\log n\left(T_{key}+T_{Dsign}+T_{Dverify}\right)+T_{sum}$, exhibiting logarithmic growth with the number of ring members. To facilitate performance comparison, Table 3 compares the signature generation steps of other literature algorithms are analogous to $T_{key}$, $T_{Dsign}$, $T_{Dverify}$, and $T_{sum}$.

The verification time mainly consists of the root node verification stage and the signature fragment verification stage in Algorithm 3. Algorithm analysis shows that the root node verification stage consumes time $T_{sum}$, while the signature fragment verification stage consumes time $T_{Dverify}$. Hence, the total verification time is $T_{verify}=\log n\cdot T_{Dverify}+T_{sum}$, also increasing logarithmically with the number of ring members. To facilitate performance comparison, Table 4 compares the signature verification steps of other literature algorithms are analogous to $T_{key}$, $T_{Dsign}$, $T_{Dverify}$, and $T_{sum}$.

As shown in Table 3 and Table 4, a comparative analysis of signature generation time, signature verification time, underlying hardness assumptions, and quantum attack resistance is provided. Compared with Reference [6], the LAPQ-LRS scheme demonstrates superior efficiency in both signature generation and verification. This advantage stems from the generally higher computational complexity of the bilinear Diffie–Hellman inverse problem employed in Reference [6] relative to the MLWE/SIS problems utilized in our scheme. When compared to lattice-based schemes in References [9,16], the LAPQ-LRS scheme achieves logarithmic growth in both signature generation and verification times as the ring size increases, resulting in significantly better scalability and efficiency for larger rings. The following section further compares the signature sizes of these schemes.

Theoretical analysis of the LAPQ-LRS scheme reveals that the signature output by the algorithm is $\sigma \left(\lambda ,s_{1},s_{2},\ldots ,s_{\left|\log n\right|+1},pk_{1},pk_{2},\ldots ,pk_{\left|\log n\right|+1}\right)$. As shown in Table 5, *n* denotes the number of ring members. Compared with Reference [6], the initial ring signature size of the proposed scheme is larger, primarily because LAPQ-LRS is based on the MLWE/SIS problems, which entail higher space complexity than the bilinear Diffie–Hellman inverse problem. However, as n increases, the logarithmic growth characteristic of LAPQ-LRS leads to a clear advantage in scalability. In comparison with References [9,16], the proposed scheme demonstrates superior performance in both signature size and growth trend under the same ring member count.

Based on the above performance analysis, the proposed scheme demonstrates comprehensive advantages in terms of signature and verification time as well as signature size, characterized by compact signature dimensions, low computational complexity, and a favorable growth trend. These attributes enable higher signing efficiency with relatively smaller signature sizes, making the scheme well-suited for practical applications. For instance, in cross-chain ring signature transaction schemes, it can reduce storage requirements on the client side and minimize latency errors through faster signing and verification processes.

### 5.2. Comparative Analysis

To provide a more comprehensive evaluation of the advancement of the proposed scheme, this section compares LAPQ-LRS with post-quantum ring signature schemes [19,20,21], which also achieve logarithmic growth. These schemes represent the state-of-the-art in achieving scalability in the field of post-quantum ring signatures.

Although the schemes mentioned above achieve theoretical logarithmic scaling, their core mechanisms generally rely on complex post-quantum zero-knowledge proof (ZKP) protocols (such as variants of the Stern protocol or lattice-based ZKPs). In contrast, the core innovation of the LAPQ-LRS scheme lies in proposing an alternative technical path: it introduces for the first time a Dilithium-based LAPQ aggregation signature operation, which functionally replaces the role of zero-knowledge proofs in constructing logarithmic-size ring signatures. The primary advantage of this design is not necessarily that it surpasses highly optimized ZKP schemes in absolute running time, but rather that it fundamentally simplifies the system architecture:

- No need to construct complex circuits: The scheme completely avoids the most complex step of designing ZKP circuits for specific lattice problems. The entire signing process is composed of a standard Dilithium signing, verification steps, and deterministic aggregation operations, resulting in a clear, modular structure;
- Significantly reduced implementation complexity and risk: Building secure and efficient post-quantum ZKP circuits is highly challenging and prone to introducing implementation vulnerabilities. LAPQ-LRS is built on mature, NIST-standardized algorithms, and all its operations (including the newly added aggregation) can be implemented using existing, widely reviewed cryptographic libraries, greatly lowering engineering difficulty and security auditing costs;
- Predictable performance and ease of optimization: The performance of the scheme is directly determined by the efficiency of the underlying Dilithium algorithm and the simple algebraic operations of the aggregation, avoiding the unpredictable performance overhead caused by circuit complexity in ZKP protocols and making system-level performance analysis and optimization more straightforward.

To clearly illustrate the differences between these technical approaches, Table 6 compares them across dimensions such as design paradigm, underlying primitive, core scaling technique, dependence on ZKP/circuits, and implementation characteristics.

As shown in Table 6, the most fundamental distinction between LAPQ-LRS and existing logarithmic schemes lies in the choice of technical path. Existing schemes adopt the “Merkle tree + ZKP” paradigm to achieve anonymity and scaling, whereas the present scheme introduces a new “Merkle tree + aggregation signature” paradigm. The core value of the latter is that it provides an implementation approach that does not rely on constructing complex ZKP circuits, making it simpler and more comprehensible. This design gives LAPQ-LRS significant advantages in terms of implementation security, code maintainability, and lowering the engineering barrier, offering a more pragmatic and risk-controllable technical path for the practical deployment of post-quantum ring signatures.

Although LAPQ-LRS avoids the construction of complex ZKP circuits through aggregation operations, achieving simplification in both structure and implementation, this design choice also introduces its own trade-offs, which are primarily reflected in the following two aspects:

- Data dependency: The aggregation process of the scheme requires synchronous acquisition of the public-key information of sibling nodes, which implies a strong requirement for the immediate availability of public-key data when constructing the signing path. This makes the scheme more suitable for scenarios where the public-key set is stable and easily accessible (e.g., registered blockchain addresses). In contrast, ZKP-based schemes may exhibit lower dependence on online data during signature generation;
- Concentration of security assumptions: The security of the scheme is entirely attributed to the MLWE/MSIS assumptions of the underlying Dilithium algorithm and the security proof of the LAPQ aggregation primitive. This simplicity means that its security boundaries are very clear, but it also places all security reliance on the robustness of this primitive, necessitating ongoing rigorous auditing.

In summary, at the cost of requiring data availability and concentrating security assumptions, LAPQ-LRS achieves significant simplification in engineering implementation and predictable efficiency, offering a more pragmatic path that is easier to deploy and verify for post-quantum ring signatures.

### 5.3. Experimental Testing

To evaluate the performance of the LAPQ-LRS schemes, the testing platform utilized an 8-core, 16-thread Intel(R) Core(TM) i7-7820X processor with 64 GB of RAM, running the Windows 10 operating system. The testing software employed was PyCharm 2021.1.1 with Python 3.9. The experiments involved multiple test runs with ring sizes of [8, 16, 32, 64, 256, 512, 1024] users to collect data on signature time, verification time, and signature size for the LAPQ-LRS scheme, and the schemes referenced in the literature.

**Figure 3:**
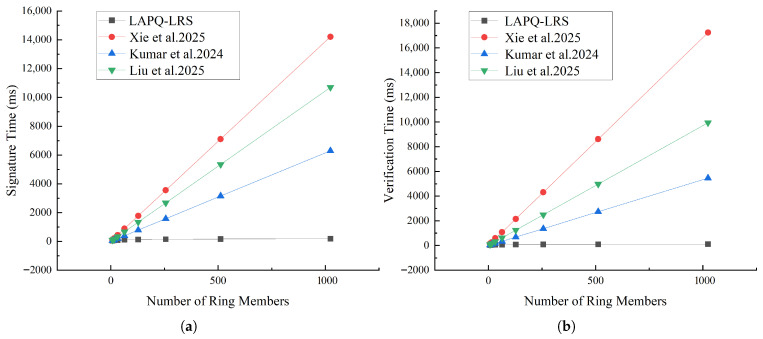
Comparative analysis of signing time and verification time. Comparative analysis of signing time (**a**) and verification time (**b**) [6,9,16].

As shown in Figure 3 and Table 7 and Table 8, as the number of ring members gradually increases, the LAPQ-LRS scheme demonstrates increasingly greater advantages over the schemes in [6,9,16] in terms of both signing time and verification time. The signing and verification times of [6,9,16] exhibit linear growth, consistent with their theoretical complexity $O\left(n\right)$. In contrast, the signing time of LAPQ-LRS grows very slowly and remains essentially stable, visually confirming its theoretical logarithmic complexity analysis $O\left(\log n\right)$. When the ring size reaches 1024 members, the signing time of LAPQ-LRS is only 184.64 ms, while that of scheme [16] is 10,699.39 ms—a difference of nearly 58 times. This gap further widens as *n* increases, reflecting the decisive advantage of logarithmic schemes in large-scale applications.

**Figure 4:**
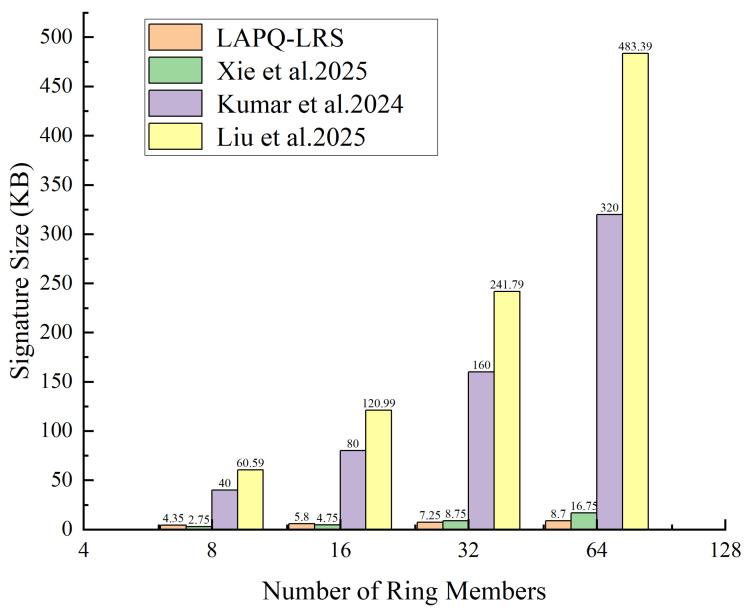
Signature size comparison [6,9,16].

Signature size is a key metric for evaluating the practicality of ring signature schemes, directly affecting communication overhead and storage costs. To more intuitively demonstrate the core contribution of the LAPQ-LRS scheme in terms of signature size, we have plotted a comparison graph (Figure 4) showing how signature size varies with the number of ring members. The graph presents measured data from each scheme at certain scales, allowing observation of the initial trends and crossover points.

As shown in Figure 4, the horizontal axis represents the number of ring members, and the vertical axis represents the signature size. The four curves represent the performance of the four schemes—LAPQ-LRS, [6,9,16]—at small scales. When the number of ring members is small (n<16), the signature size of LAPQ-LRS is slightly larger than that of [6], which stems from the inherently larger public-key size of lattice-based cryptographic primitives. However, at n=32, the signature size of LAPQ-LRS (7.25 KB) already falls below that of the other schemes, and as n increases, its advantage begins to emerge. At n=64, LAPQ-LRS (8.7 KB) becomes significantly smaller than [16] (483.39 KB).

To more intuitively demonstrate the performance improvement of the proposed scheme over the existing Dilithium-based scheme [16], Table 9 calculates the relative reduction percentages of LAPQ-LRS compared to [16] across three key metrics—signing time, verification time, and signature size—under different ring sizes. The calculation formula is:

$$\mathrm{Improvement}\;\mathrm{Percentage}=\left(1-\frac{\mathrm{LAPQ-LRS}\;\mathrm{Performance}\;\mathrm{Metric}}{\;\mathrm{Performance}\;\mathrm{Metric}}\right)\times 100\%$$

“Performance Metric” refers to the value of signing time, verification time, or signature size obtained from experimental results under the same ring-size setting. The corresponding values from references [16] are taken from its reported results under identical ring sizes.

As shown in Table 9, the performance advantages of the LAPQ-LRS scheme grow dramatically as the ring size increases, demonstrating a clear logarithmic scaling advantage. When the number of ring members reaches 1024, signing time is reduced by 98.25%, verification time by 98.90%, and signature size by 99.81%, providing complete validation data and trend illustration. This table strongly confirms that, compared to traditional linear schemes, LAPQ-LRS possesses overwhelming efficiency and space advantages in large-scale application scenarios.

The core contribution of this scheme lies in being the first to combine aggregation signature operations with a Merkle tree structure, achieving logarithmic growth in both time and space complexity for post-quantum ring signatures without relying on complex zero-knowledge proof circuits. The experimental results and theoretical analysis provide robust evidence for this contribution.

## 6. Conclusions

To address the vulnerability of traditional mathematical hard problem-based linkable ring signatures against quantum attacks, as well as the issues of large signature sizes and high circuit complexity caused by post-quantum zero-knowledge proof protocols in existing post-quantum ring signature schemes, this paper proposes a logarithmic-size post-quantum linkable ring signature scheme based on aggregation operations, built upon the NIST-standardized post-quantum digital signature algorithm Dilithium. The scheme constructs a Merkle tree from ring members’ public keys using a hash algorithm to achieve logarithmic-scale signing and verification. For the first time, a Dilithium-based aggregation operation is introduced to replace zero-knowledge proof protocols, effectively avoiding complex circuit structures. Correctness analysis demonstrates that the LAPQ-LRS scheme satisfies the required correctness properties. Security analysis under the random oracle model proves that the scheme provides anonymity, unforgeability, and linkability. Comparative experimental evaluations show that the proposed scheme significantly reduces signing and verification times compared to other quantum-attack-resistant ring signature schemes. In terms of signature size, the LAPQ-LRS scheme also exhibits advantages, with signatures growing logarithmically. However, since the LAPQ-LRS scheme is designed based on lattice-based hard problems, it entails higher space complexity, and the aggregation process requires more time than the original Dilithium scheme. Future work will focus on optimizing the signature and verification algorithms to improve operational efficiency and further refine the scheme.

## Figures and Tables

**Table 1:** Comparison of major post-quantum ring signature schemes.

Scheme	Methodology	Main Contribution	Limitation
[13]	Lattice-based construction	First on-chain linkable scheme	Linear complexity inefficient
[14]	Identity-based lattice construction	Identity-based key simplification	linear growth
[16]	Dilithium-based approach	Traceable scheme optimization	Large signature size
[19]	Zero-knowledge proof (ZKP)	Logarithmic threshold ring signature	Complex circuit construction
[20]	Merkle tree + ZKP	Logarithmic deniable signature	High proof generation overhead
[21]	NTRU lattice + ZKP	Identity-based ring signcryption	Dependent on ZKP protocols

**Table 2:** Parameter and function definitions.

Parameters	Definitions
*R*	Polynomial ring Zxxxg+1xg+1
$R_{q}$	Polynomial ring modulo *q* Zqxxxg+1xg+1
*g*	Ring dimension: 256
*n*	Ring size
*q*	Modulus $q=2^{23}-2^{13}+1$ = 8,380,417
*d*	High/low-bit separation precision *d*: 13
k,l	Matrix dimensions in Dilithium2: k=4,l=4
η	Secret coefficient bound η=2
τ	Challenge weight τ=39
$\gamma_{1},\gamma_{2}$	$\gamma_{1}=2^{17}$,γ2=γ1−βγ1−β22
β	Rejection bound β=τ·η
$S_{\eta }^{k}$	Element in a ring

**Table 3:** Comparison of signature generation time overheads and hardness assumptions.

Scheme	Signature Generation Time	Hardness Assumption
[6]	$\left(3n-2\right)\cdot T_{Dsign}+T_{key}$	Bilinear Diffie-Hellman Inversion
[9]	$\left(3+n\right)\cdot T_{Dsign}$	SIS
[16]	$2n\cdot T_{Dsign}+T_{key}$	MLWE/SIS
LAPQ-LRS	$\log n\cdot \left(T_{key}+T_{Dsign}+T_{Dverify}\right)+T_{sum}$	MLWE/SIS

**Table 4:** Comparison of signature verification time overheads and hardness assumptions.

Scheme	Signature Verification Time	Hardness Assumption
[6]	$3n\cdot T_{Dverify}+2T_{key}$	Bilinear Diffie-Hellman Inversion
[9]	$n\cdot T_{Dverify}$	SIS
[16]	$2n\cdot T_{Dverify}+T_{key}$	MLWE/SIS
LAPQ-LRS	$\log n\cdot T_{Dverify}+T_{sum}$	MLWE/SIS

**Table 5:** Signature size comparison (KB) and hardness assumptions.

Scheme	Signature Size	Hardness Assumption
[6]	0.25·n+0.75	Bilinear Diffie-Hellman Inversion
[9]	5.00·n	SIS
[16]	7.55n+0.19	MLWE/SIS
LAPQ-LRS	1.45·logn	MLWE/SIS

**Table 6:** Comparison of technical approaches with logarithmic post-quantum ring signature schemes.

Comparison Dimension	[19]	[20]	[21]	LAPQ-LRS
Signature Type	Linkable Threshold Ring Signature	Non-interactive Deniable Ring Signature	Identity-Based Linkable Ring Signcryption	Linkable Ring Signature
Underlying Hard Problem	SIS/LWE	SIS/LWE	NTRU-SIS	MLWE/MSIS (Dilithium)
Logarithmic Realization Technique	Merkle Tree + Stern-like ZKP	Merkle Tree + ZKP	Merkle Tree + ZKP & Commitments	Merkle Tree + Aggregation Signature (LAPQ)
Dependence on ZKP/Circuits	Yes (requires ZKP circuit)	Yes (requires ZKP circuit)	Yes (requires ZKP circuit)	No
Main Technical Contribution	Threshold integration, logarithmic size	Achieves deniability	Identity-based and signcryption	“Aggregation-instead-of-proof” paradigm
Implementation Complexity & Risk	High (complex ZKP circuits)	High (complex ZKP circuits)	High (NTRU + ZKP circuits)	Low (extends standard primitives)
Primary Performance Determinant	ZKP circuit efficiency, rounds	ZKP proof generation	NTRU operations and ZKP circuits	Base signature efficiency and aggregation

**Table 7:** Test results of signature generation time for different algorithms (ms).

Scheme	8	16	32	64	128	256	512	1024
[6]	106.32	217.87	440.88	887.06	1776.46	3550.24	7105.37	14,207.96
[9]	50.32	98.56	194.05	392.47	786.41	1574.38	3147.23	6293.34
[16]	84.68	168.12	335.27	668.76	1335.56	2672.76	5348.58	10,699.39
LAPQ-LRS	54.79	73.34	90.61	110.42	128.99	147.52	166.09	184.64

**Table 8:** Test results of signature verification time for different algorithms (ms).

Scheme	8	16	32	64	128	256	512	1024
[6]	151.78	264.03	588.26	1077.46	2151.94	4307.85	8615.76	17,241.17
[9]	43.92	85.74	166.67	341.49	687.82	1361.52	2733.72	5461.84
[16]	78.41	156.25	311.93	623.29	1244.67	2485.08	4972.51	9946.79
LAPQ-LRS	30.69	41.75	54.37	66.43	77.12	87.35	98.74	109.26

**Table 9:** Performance improvement percentage of LAPQ-LRS compared to ccheme [16].

Ring Size (n)	Signing Time Reduction	Verification Time Reduction	Signature Size Reduction (Theoretical)
8	35.33%	60.86%	51.38%
16	56.36%	73.28%	67.64%
32	72.98%	82.57%	77.48%
64	83.49%	89.34%	83.58%
128	90.34%	93.80%	88.28%
256	94.48%	96.49%	91.60%
512	96.90%	98.02%	94.04%
1024	98.25%	98.90%	99.81%

## Data Availability

The code employed in this study was innovatively adapted from the open-source Dilithium project available on GitHub, https://github.com/GiacomoPope/dilithium-py, access date: 11 December 2025. Due to privacy considerations, the complete modified source code is not publicly archived. However, the detailed pseudocode provided within the article is sufficient to ensure the correct replication of the scheme. The corresponding data can be generated accordingly, thereby confirming the availability of the research outcomes and methodology.
